# Reviewer acknowledgement 2013

**DOI:** 10.1186/1476-0711-13-8

**Published:** 2014-02-21

**Authors:** Hakan Leblebicioglu

**Affiliations:** 1 Department of Infectious Diseases and Clinical Microbiology, Faculty of Medicine, Ondokuz Mayis University, Samsun, Turkey

## Abstract

**Contributing reviewers:**

*Annals of Clinical Microbiology and Antimicrobials* would like to thank the following colleagues for their assistance with peer review of manuscripts for the journal in 2013.

## 

Arturo Abdelnour

Costa Rica

Esragül Akinci

Turkey

Serife Akalin

Turkey

Patrick Eberechi Akpaka

Trinidad and Tobago

Ali Al-Ahmad

Germany

Leopoldina Almeida

Brazil

Mustafa Altindis

Turkey

Seza Arslan

Turkey

Cenk Aypak

Turkey

Cecile Badet

France

Vasudev Ballal

India

John Beier

United States of America

Ria Benko

Hungary

Mehlika Benli

Turkey

Ramesh Bhat. Y

India

Blasé Billack

United States of America

Jorge Blanco

Spain

Juergen Bohnert

Germany

Rana Bonds

United States of America

Jose Bordon

United States of America

Michael Borg

Malta

Esperanza Cabrera

Philippines

Emmanuelle Cambau

France

Karyne Carvalho

Brazil

Benedetto Maurizio Celesia

Italy

Salih Cesur

Turkey

Shivendra Chaurasiya

India

Nicole Christian

Jamaica

Sasitorn Chusri

Thailand

Patricia Cisalpino

Brazil

Paul Cook

United States of America

Omer Coskun

Turkey

Asta Dambrauskiene

Lithuania

Michael David

United States of America

Alessandro De Logu

Italy

Beryl De Souza

United Kingdom

Chris Del Mar

Australia

Tuna Demirdal

Turkey

Cicero Dias

Brazil

Ermias Diro

Ethiopia

Murat Dizbay

Turkey

Veeramuthu Duraipandiyan

India

Hakan Erdem

Turkey

Ilknur Erdem

Turkey

Alper Ergin

Turkey

Hatice Ertabaklar

Turkey

Saban Esen

Turkey

Özgen Eser

Tuvalu

Benjamin Evans

United Kingdom

Mohammad Feizabadi

Iran

Judith Flanagan

Australia

Serap Gencer

Turkey

Ertugrul Guclu

Turkey

Hanefi Cem Gul

Turkey

Azar Hadadi

Iran

Christopher Harrison

United States of America

Elizabeth Hirsch

United States of America

Salih Hosoglu

Turkey

Duanen Hospenthal

United States of America

Elias Losifidis

Greece

Miro Islamic

Egypt

George Jacoby

United States of America

Amita Jain

India

Wafaa Jamal

Kuwait

Judy Natalia Jiménez Q

Colombia

Cheol-In Kang

South Korea

Oguz Karabay

Turkey

Aynur Karadenizli

Turkey

Afework Kassu

Ethiopia

Esra Kocoglu

Turkey

Tse Hsien Koh

Singapore

Tse Hsien Koh

China

Mehmet Koroglu

Turkey

Volkan Korten

Turkey

Andrea Kwa

Singapore

Cesar Lau-Cam

United States of America

Adamantia Liapikou

Greece

Julianne Lockwood

United Kingdom

Yolanda López-Vidal

Mexico

Hongxiang Lou

China

Eric Macy

United States of America

Jane Marsh

United States of America

Eric Maury

France

Ahmet Mavi

Turkey

Sonia Melino

Italy

Athanasios Michos

Greece

Maria Leticia Miranda Fernandes Estevinho

Portugal

Yoko Miyasaki

United States of America

Kevin Nash

United States of America

Scott Newsome

United States of America

Angela Novais

Portugal

David Ogbolu

Nigeria

Aziz Ogutlu

Turkey

Oral Oncul

Turkey

Tanja Opriessnig

United States of America

Resar Ozaras

Turkey

Jorge Paez

Brazil

Jiachuan Pan

United States of America

Jigensh Patel

United States of America

Duygu Percin

Turkey

Laurent Poirel

Switzerland

Ryan Quock

United States of America

Swaraj Rajkhowa

India

Meenakshi Rana

United States of America

Meher Rizvi

India

Jose Manuel Rodriguez Martinez

France

Horieh Saderi

Iran

Caroline Schneeberger

Netherlands

Abiola Senok

Saudi Arabia

Yan Shi

China

Bidya Shrestha

Nepal

Margrete Solheim

Norway

Julián Solís García del Pozo

Spain

Vijaya Bharathi Srinivasan

India

Olgica Stefanovic

Serbia

Thomas Stroem

Denmark

Wei-Juin Su

Taiwan

Hiromichi Suzuki

Japan

Samuel Taiwo

Nigeria

Thean Yen Tan

Singapore

Benjamin Tanner

United States of America

Meltem Tasbakan

Turkey

Hare Krishna Tiwari

India

Greg Tyrrell

Canada

Aysel Ugur

Turkey

Orhan Ünal

Turkey

Canan Usta

Turkey

Cemal Üstün

Turkey

Haluk Vahaboglu

Turkey

Gustavo Varela

Uruguay

Ralf-Peter Vonberg

Germany

Lei Wang

United States of America

Nele Wellinghausen

Germany

Mark Wolff

United States of America

Yan Xu

China

Hava Yilmaz

Turkey

Aysegul Yagci

Turkey

Hideki Yamamura

Japan

Reza Yousefi-Nooraie

Canada

Ayse Yuce

Turkey

Martin Zabka

Czech Republic

Esther Zander

Germany

Qiqing Zhang

China

Guang-Rong Zhao

China

Dalian Zhong

United States of America

